# The cost effectiveness of a quality improvement program to reduce maternal and fetal mortality in a regional referral hospital in Accra, Ghana

**DOI:** 10.1371/journal.pone.0180929

**Published:** 2017-07-14

**Authors:** David M. Goodman, Rohit Ramaswamy, Marc Jeuland, Emmanuel K. Srofenyoh, Cyril M. Engmann, Adeyemi J. Olufolabi, Medge D. Owen

**Affiliations:** 1 Hubert-Yeargan Center for Global Health, Department of Obstetrics and Gynecology, Duke University Medical Center, Durham, NC, United States of America; 2 Gillings School of Public Health, University of North Carolina, Chapel Hill, NC, United States of America; 3 Sanford School of Public Policy & Duke Global Health Institute, Duke University, Durham, NC, United States of America; 4 Ridge Regional Hospital, Ghana Health Service, Accra, Ghana; 5 Department of Pediatrics, University of Washington & Seattle Children’s Hospital, Seattle, WA, United States of America; 6 Department of Anesthesiology, Duke University Medical Center, Durham, NC, United States of America; 7 Department of Anesthesiology, Wake Forest School of Medicine, Winston-Salem, NC, United States of America; University of Maryland School of Medicine, UNITED STATES

## Abstract

**Objective:**

To evaluate the cost-effectiveness of a quality improvement intervention aimed at reducing maternal and fetal mortality in Accra, Ghana.

**Design:**

Quasi-experimental, time-sequence intervention, retrospective cost-effectiveness analysis.

**Methods:**

Data were collected on the cost and outcomes of a 5-year Kybele-Ghana Health Service Quality Improvement (QI) intervention conducted at Ridge Regional Hospital, a tertiary referral center in Accra, Ghana, focused on systems, personnel, and communication. Maternal deaths prevented were estimated comparing observed rates with counterfactual projections of maternal mortality and case-fatality rates for hypertensive disorders of pregnancy and obstetric hemorrhage. Stillbirths prevented were estimated based on counterfactual estimates of stillbirth rates. Cost-effectiveness was then calculated using estimated disability-adjusted life years averted and subjected to Monte Carlo and one-way sensitivity analyses to test the importance of assumptions inherent in the calculations.

**Main outcome measure:**

Incremental Cost-effectiveness ratio (ICER), which represents the cost per disability-adjusted life-year (DALY) averted by the intervention compared to a model counterfactual.

**Results:**

From 2007–2011, 39,234 deliveries were affected by the QI intervention implemented at Ridge Regional Hospital. The total budget for the program was $2,363,100. Based on program estimates, 236 (±5) maternal deaths and 129 (±13) intrapartum stillbirths were averted (14,876 DALYs), implying an ICER of $158 ($129-$195) USD. This value is well below the highly cost-effective threshold of $1268 USD. Sensitivity analysis considered DALY calculation methods, and yearly prevalence of risk factors and case fatality rates. In each of these analyses, the program remained highly cost-effective with an ICER ranging from $97-$218

**Conclusion:**

QI interventions to reduce maternal and fetal mortality in low resource settings can be highly cost effective. Cost-effectiveness analysis is feasible and should regularly be conducted to encourage fiscal responsibility in the pursuit of improved maternal and child health.

## Introduction

Despite recent and historic global decreases in maternal and child mortality during the Millennium Development Goals (MDGs) era, this global reduction masks immense regional variation, and the prevention of maternal death continues to be a high-priority for national governments and the public health community. In 2015, over 300,000 maternal deaths occurred worldwide; 201,000 (66%) of these occurred in sub-Saharan Africa (SSA).[1] Within SSA, maternal mortality ratios remain fifty times higher than in high-income countries.[1] Meanwhile, stillbirths remain a silent and devastating problem. Each year, 2.6 million stillbirths occur; 1.2 million of these lives are lost intrapartum. In SSA, stillbirth rates are 8 times those of high-income settings; given current trends, it will take 160 years for stillbirth rates in SSA to fall to the levels found in high-income countries.[2]

Providing maternal and fetal health (MFH) in low- and middle-income countries (LMICs) requires a coordinated effort of antenatal care, delivery of skilled services, and emergency obstetric care, and depends on overcoming demand-side and supply-side barriers to quality care. In the context of MFH, demand-side barriers include common obstacles to accessing care such as finanicial limitations, transportation challenges, and insufficient patient education.[3] A thorough discussion on efforts to improve demand-side financing is available from the WHO.[4] Recent successful examples include the expansive incentive-based Janani Suraksha Yojana program in India[5], a voucher-scheme in Uganda[6], and maternal insurance in Nigeria[7]. These programs report incremental cost-effectiveness ratios (ICER) of $46-$302 per disability-adjusted life-year (DALY) averted and have been deemed highly cost effective.

As governments and non-governmental organizations (NGOs) work to address demand-side barriers, more deliveries will be shifted towards institutions that will need to prepare for the increased number of deliveries. Supply-side interventions will need to address the care delivered by the health system and include the actions of providers and the environment in which care is given. For example, it is known that 15% of pregnancies require advanced emergency treatment for complications that are often difficult to predict or prevent.[8,9] Unfortunately, many low-resource comprehensive emergency obstetric care CEmOC facilities cannot deliver high quality emergency care and are not prepared for the shift towards greater institutional delivery.[10–12] Barriers to its provision include a lack of leadership and patient centeredness, absence of resources, and poor operational systems.[10–14] These deficiencies in turn lead to inadequate management of complex obstetric cases and untimely death, and underscore the need for a comprehensive approach that would strengthen the capacity of high-risk referral centers.

Improving CEmOC requires careful analysis and trade-offs, especially in resource-limited countries such as Ghana where maternal mortality and stillbirth rates remain high, and public health resources limited. Yet there is a dearth of empirical evidence regarding the impact and cost-effectiveness of CEmOC capacity-building efforts. Some argue that CEmOC interventions are more costly than other measures to reduce maternal and neonatal death, and require expertise that is mostly unavailable in low income countries.[15,16] Others report a lack of evidence on the disease burden, cost, and effectiveness of intervention packages aimed at referral-level facilities.[17] Responding to such arguments, the WHO Executive Board has recognized the need to enhance international cooperation, institutional and operational capacity, and infrastructure for public health.[18]

Indeed, there are few if any retrospective cost-effectiveness analyses of quality improvement (QI) programs aimed at improving CEmOC in low-resource settings, although some studies provide insights about other CEmOC interventions. Broughton and co-authors describe a successful partnership with the Ministry of Health in Niger that reduced mortality by focusing on active management of the third stage of labour and immediate essential newborn care. They report an ICER of $291 in 2015 USD.[19] In 2003, McCord et al. reported on an intervention using local surgeons to train local general physicians to perform life-saving surgeries including cesarean sections in a small hospital in Bangladesh at a cost of $57 per DALY [20]. Also in 2003, a summary of the Save the Mothers Initiative, a Uganda-Canada collaboration, described the costs of an intervention to increase the availability and utilization of CEmOC services, without commenting on its effects.[21] In Mozambique, a partnership between the University of Michigan and the Instituto Superior de Ciencias de Saude showed that training surgical technicians was cost-effective at $39 per major surgery compared with $144 for surgeons and obstetricians.[22]

It is more common in the MFH literature to use prospective modelling to estimate the cost-effectiveness of potential interventions. Prospective studies have been important for policy advocacy related to MFH interventions, as they routinely show that such investment is beneficial for society. Alkire et al. estimated that a program to reduce obstetric fistula by providing access to cesarean delivery would have an ICER of $277 in Ghana.[23] Adam et al. analyzed effectiveness data from several sources and calculated an ICER of $270 (2015 USD) for intervention packages that encompassed CEmOC in SSA.[17] Erim et al. modeled the cost-effectiveness of MFH interventions in Nigeria. They emphasized the need to couple cost-effective family planning access and contraception if maternal mortality targets are to be reached, given that “there is a threshold above which further reductions in mortality…are not possible” without high-quality CEmOC. [24] Helping tertiary referral obstetric hospitals in low-resource countries to provide high-quality care may be feasible if QI methodologies used more frequently in high-resource hospitals are also introduced using coaching and consistent monitoring. Unfortunately, to the best of our knowledge, there are no published analyses that assess the question of whether such QI interventions are cost effective.

This study provides evidence that helps to address this knowledge gap, focusing on a five-year intervention that aimed to reduce maternal and fetal mortality in Accra, Ghana. Kybele, an international NGO that promotes safe childbirth through innovative partnerships, worked with the Ghana Health Service (GHS) to improve CEmOC at a large urban referral hospital in Accra. The intervention comprised education, leadership development, systems strengthening, and QI.[25, 26] As discussed elsewhere, the program proved highly effective at reducing maternal and fetal mortality rates.[14, 27] Still, it is important to recognize that not every change is an improvement[28]), and not every improvement is cost-effective. In order to be accountable to the GHS and other donors, we therefore present a CEA of the intervention which should help direct future efforts to build on this program, as well as the work of other similar organizations.

## Methods

This study considers the cost effectiveness of a five-year collaboration between Kybele, Inc.[29] and the GHS at the Ridge Regional Hospital (RRH) in Accra, Ghana (January 2007- December 2011). RRH is the highest-volume obstetric referral center within the GHS with a 90-bed maternity unit providing CEmOC. Approximately 70% of deliveries at RRH are referrals from other hospitals and polyclinics located in and around Accra. In 2004, RRH experienced 2,000 deliveries per year that rose to 4,793 deliveries in 2006 (institutional reports, not published). By the end of 201 when the intervention ended, RRH managed 9,357 deliveries. This 95% increase in patient volume over the course of the collaboration was a result of demand-side incentives in Ghana, changes in the referral patterns in Accra, and recognition of improving quality by the community.[25, 27]

Based on previous work of Kybele, the GHS invited the organization to lead a QI program and build up provider capacity for leading other similar interventions in the future. The overall aim of the partnership was to reduce the maternal mortality ratio (MMR, defined as maternal deaths per 100,000 live births) and stillbirth rates (SBR, defined as intrapartum stillbirths per 1,000 live births) by 50% and to establish an “Obstetric Center of Excellence” at RRH[25].

**Fig 1:**
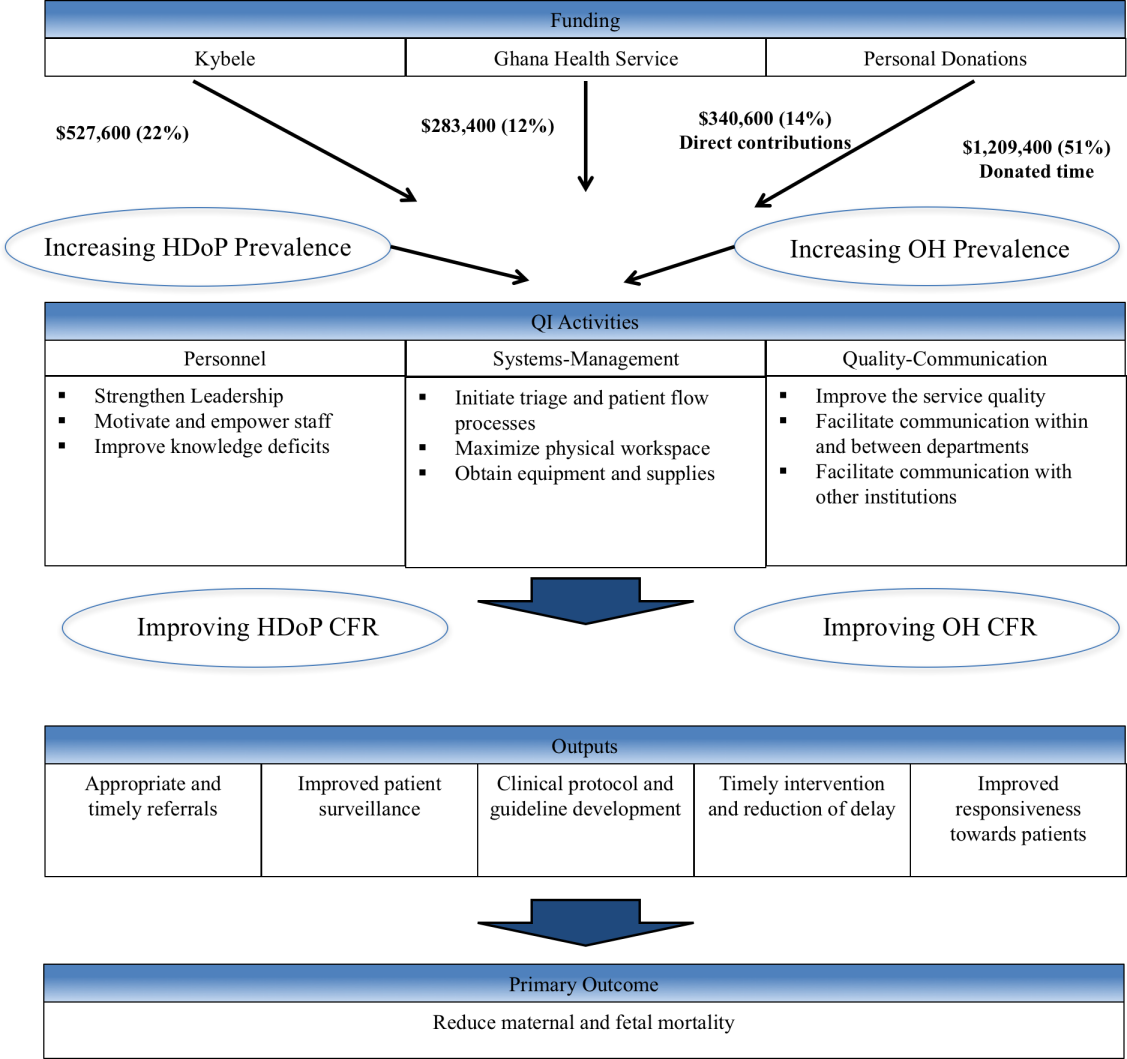
Analytical model for Kybele-GHS institutional framework of change model. GHS, Ghana Health Service; HDoP, hypertensive disorders of pregnancy; OH, obstetric hemorrhage; CFR, case fatality rate; QI, quality improvement; Dollar values in $2015 USD.

Resources were not available to build a new facility or to significantly increase staff in the midst of the changes in demand for services at RRH. Instead, volunteers from high-volume obstetric departments in the United States and United Kingdom made triannual visits to Ghana to coach and mentor providers and administrators at RRH to optimize the protocols and processes through systematic QI. The RRH-Kybele team jointly identified deficiencies in delivery of care, and developed solutions guided by a strategic plan. The plan specified 97 improvement activities categorized into three bundles based on personnel, systems management, and quality communication.[27] The hypothesis was that long-term investment would lead to a strengthened system that would display stepwise improvement leading to a reduction in maternal and fetal mortality (Fig 1). Institutional review board approval was granted by Wake Forest University and the GHS for the conduct of the work.

A complete analysis of the changes over time has been published elsewhere along with more descriptive supplemental material.[27] The Kybele-GHS partnership is unique in that it did not focus primarily on providing materials commonly associated with healthcare costs, such as medications and supplies. The partnership instead worked primarily to identify gaps and create processes that enabled the staff to use their resources more effectively. Of the 97 processes that were designed, the mean implementation was rate 68%, and 26 processes were found to be positively correlated with mortality reduction.[15] Activities included developing leadership capabilities, facilitating communication, and improving timeliness of care. For example, morning meetings between doctors, house officers, and midwives were organized to allow daily discussion of challenges and successes.[14] Redesigning the workflow to create a triage process similarly did not require hiring new staff, but rather facilitated more efficient labor allocation, and improved safety for women.

### Description of program costs

Program costs were prospectively collected and tracked the multiple sources of financing of the intervention. All dollar values presented in this study were adjusted for inflation and standardized to 2015 USD. Kybele as an organization covered its own administration, logistics, and educational materials. Members of Kybele–practicing professionals from North America and England with backgrounds leading high-quality teams in obstetrics, midwifery, anesthesiology, neonatology, and public health–provided voluntary (uncompensated) services. The duration of each trip was five to 12 days. Individual participants provided their own funding for airfare, visas, and other expenses associated with travel to Ghana; these costs were included in our cost analysis. The main intervention trips were triannual, although occasional shorter trips were made for special meetings with the GHS. The GHS provided in-country accommodation including housing, meals, and transportation for volunteers and arranged medical privileges.

A detailed project budget was kept throughout the course of the intervention and is shown in Table 1, organized by organization. Eighty-six professionals traveled to Ghana 176 times; 34 of whom (40%) returned multiple times. The value of volunteered professional time was included although it was not money actually spent. This represents the opportunity cost of the professionals offering their services within their home institutions. Values were determined based on the U.S. Department of Labor, National Occupational Employment and Wage Estimates, Role and Occupation Code.[30] Team members’ time contributions were assigned as follows based on 10-hour work days: physicians-$1100/day, nurse anesthetist $750/day, midwife/engineer/nurse practitioner/consultants $500/day, nurse/resident/biostatistician $330/day. Assigning value to volunteer time is challenging, and therefore was subjected to 25% variation in sensitivity analyses. Twelve Ghanaians were also sponsored to visit the US or England for short-term hospital observations with costs primarily incurred by Kybele. Triannual mortality conferences were organized during the program that improved communication between GHS, RRH, and referring hospitals. The value of Ghanaian attendees’ time at meetings and for other meeting costs totaled $91,670.

**Table 1:** Kybele-GHS partnership budget.

	Kybele	Participant	GHS	Time Value	Total Cost
**Jan-07**	$4,120	$29,310	$8,240	$135,910	$177,600
**Mar-07**	$0	$2,290	$0	$6,300	$8,600
**Jun-07**	$340	$18,430	$2,180	$28,970	$49,900
**Jan-08**	$880	$21,710	$7,930	$103,150	$133,700
**Mar-08**	$3,530	$8,380	$1,210	$7,270	$20,400
**May-08**	$330	$7,270	$2,200	$11,680	$21,500
**Sep-08**	$6,940	$16,750	$1,980	$53,120	$78,800
**Jan-09**	$3,100	$22,890	$2,770	$97,770	$126,500
**May-09**	$770	$14,600	$1,660	$36,500	$53,500
**Sep-09**	$6,300	$28,420	$4,090	$67,240	$106,100
**Jan-10**	$4,460	$34,920	$8,700	$138,390	$186,500
**May-10**	$3,370	$27,090	$2,830	$75,720	$109,000
**Sep-10**	$4,130	$19,800	$4,460	$79,420	$107,800
**Jan-11**	$2,430	$29,750	$6,960	$153,710	$192,900
**May-11**	$9,810	$26,060	$6,120	$65,300	$107,300
**Aug-11**	$3,060	$0	$0	$5,800	$8,900
**Sep-11**	$9,920	$31,230	$9,500	$143,160	$193,800
**US/UK Visits**	$13,740	$1,650	$0	$0	$15,400
**Conference/Meals**	$0	$0	$91,670	$0	$91,700
**CAPITAL**	$452,320	$0	$120,900	$0	$573,200
**TOTAL**	$527,600	$340,600	$283,400	$1,209,400	$2,363,100
**Percentage**	22%	14%	12%	51%	100%

Capital was invested by the GHS including equipment purchases and minor renovations amounting to $120,910. Half of this investment was for beds that had depreciated by the end of the intervention. The other investments included painting of walls, hanging of privacy curtains, and repairing of floors in the labour ward. In 2008, 2009, and 2011, Kybele provided refurbished equipment to the hospital, including two ultrasound machines, fetal monitors, blood pressure monitors, anesthesia and theatre equipment, along with a variety of smaller medical devices and supplies amounting to $452,320. These donations were either depreciated at the time of donation or consumables that were not subject to annualization. Midwives were posted to RRH during this time, but the hires were proportional to the delivery volume and did not make up for the chronically low staff ratio of 6.6–7.1 midwives per 1,000 deliveries.

As seen in Table 1, the value of professional time accounted for 51% of the total budget. Individual participants contributed 14% of the budget, Kybele provided 22%, and the GHS covered 12%. The total cost of the program was $2,363,100. This analysis does not consider the costs of delivering care such as changes in medication usage and inpatient length of stay, but rather focuses on the cost of delivering the program. There was no budget for increasing delivery of services, so the QI project was structured to improve care in a cost-neutral way, mainly by reducing delays and improving communication. Given that most of the funding came from international sources, purchasing-power parity adjustments were not made in order to be able to assess the cost-effectiveness relative to standard threshholds discussed in the literature.

### Estimating disability-adjusted life years (DALY) for maternal and fetal death in Ghana

The DALY is the most commonly used metric for quantifying the burden of disease in a given population in low- and middle-income countries.[31–34] The DALY indicates the number of years of healthy life lost due to death or disability. Disability-adjusted life years are the sum of the present value of future years of life lost through premature mortality (YLL) and disability (YLD), which accounts for the relative severity of mental or physical impairments that stem from the disease using a disability weight.

DALY=YLL+YLD

The Global Burden of Disease (GBD) project provides important guidance on the appropriate methods for computing DALYs, but several issues related to cost effectiveness analysis using DALYs remain controversial in the literature. In particular, the most recent editions of the GBD have not discounted future DALYs [20, 22], thereby removing assumptions that individuals and societies value years of life lost in the future at a reduced rate compared to current years of life lost. They also have not applied age weighting, which considers the value of years of life lost to vary with age. Methodologies used by the WHO have historically used both of these practices, however, on the basis of arguments made by many health economists.[34, 35] This analysis takes that controversy into account and presents the results of both methods. The methods used for discounting DALYs have been well-discussed by other authors.[32] In this study we used standard values for age weighting and discounting as seen in Table 2.

**Table 2:** Sensitivity analysis parameters.

Parameter	Low	Mid	High	Distribution of variation
**Average Age**	26.9	29.9	32.9	Uniform
**Remaining Life Expectancy**	39.1	43.4	47.7	Uniform
**Stillbirth Life Expectancy**	55.1	61.2	67.3	Uniform
**K (age weighting)**	—	1	—	Used in discounting formula
**C (constant)**	—	0.1658	—	
**r (discounting time)**	—	0.03	—	
**B (age weight function)**	—	0.04	—	
**Disability weight (discounting)**	0.2	0.3	0.4	Uniform
**Disability multiplier (M)**	1	2	3	Uniform
**Professional time**	$907,050	$1,209,400	$1,511,750	Uniform
**2008–2011 HDoP Prevalence**	-1.96*SE*	Observed yearly value	+1.96*SE	Normal
**2008–2011 OH Prevalence**	-1.96*SE	Observed yearly value	+1.96*SE	Normal
**2008–2011 HDoP CFR**	-1.96*SE	Observed yearly value	+1.96*SE	Normal
**2008–2011 OH CFR**	-1.96*SE	Observed yearly value	+1.96*SE	Normal
**2008–2011 Maternal Deaths**	-1.96*SE	Observed yearly value	+1.96*SE	Normal
**2008-2011Stillbirths**	-1.96*SE	Observed yearly value	+1.96*SE	Normal

SE: Standard error for sample

The average age of maternal death at RRH was determined to calculate DALYs. Maternal deaths were grouped in 5-year increments from <15–49 years of age, and the proportion of deaths from each age group was calculated based on the age for each maternal death observed during the intervention for 2007–2011. It was assumed that reductions in maternal deaths at RRH were proportionate for each age group. The years of life lost due to premature death were calculated using Ghana-specific life expectancy for each age group based on values that were interpolated from 2000 and 2012 standard life tables for Ghana.[36]

YLD is based upon previously described estimates that for every maternal death, 5–20 women are disabled due to complications.[23, 37–41] This ratio is represented as the disability multiplier, “M”. Maternal injury is a significant contributor to the global burden of obstetric disease that is not represented by the MMR.[42] It was assumed that the ratio of maternal deaths to maternal injuries was the same before and after the intervention and distributed proportionally across age groups. Because this study takes place at a referral center, it was determined that many of the morbidities women develop during home deliveries should not be considered. We hypothesized that proper management of obstetric hemorrhage (OH) and hypertensive disorders of pregnancy (HDoP) would help prevent morbidities such as loss of fertility due to cesarean hysterectomy and stroke due to uncontrolled hypertension and eclamptic seizures. The GBD uses a disability weight (D) of 0.3,[43] for these conditions that are assumed to last for life. This was conservatively estimated to occur with an M of 2, which indicates that for every life saved through CEmOC, improved care prevented two near-misses as well.

There is discussion about whether or not to include stillbirth in DALY calculations.[44–46] This study does not intend to resolve ethical debate about assigning value to life lost in utero, but recognizes the immense cost to families when a fetus dies during delivery. The WHO, in the 2013 Global Health Estimates, recommends that stillbirths be considered as years of life lost and bases the value on standard life tables for life expectancy at birth.[31] For obvious reasons, there is no YLD component to stillbirth. The only stillbirths considered were fresh stillbirths that occurred as a result of intrapartum complications. In this study, stillbirths were thus included in the base analysis and the sensitivity analysis.

### Estimating the number of deaths and DALYs averted through the partnership

This study compares the number of maternal deaths avoided due to the partnership intervention to a “no-intervention” counterfactual that was not actually observed, but rather estimated from the quasi-experimental pre- and post-intervention evaluation of the program. Typically, NGOs would use mortality rates as the standard measure for improvement over time. As mentioned previously, RRH experienced a period of significant growth just before and during the intervention when the volume and acuity of patients changed significantly. A full discussion of the analysis of these changes has been published previously[27], but we will summarize the salient points of the analysis here. To determine what likely would have occurred if the program had not taken place, we considered two scenarios. The first is one in which the maternal mortality ratio (MMR) was assumed to remain steady. Using this method, the baseline 2007 MMR was used to predict the number of maternal deaths that would have occurred had the intervention not been present. Any reduction in the number of maternal deaths is assumed to be an improvement over this counterfactual baseline, but attribution of causality is difficult, given that there are demand- and supply-side factors that contribute to MMR.

The second approach is to treat the case-fatality rates of different maternal complications as being in steady state, and to consider the difference between predicted deaths and observed deaths as the measure of improvement. Cases and fatalities are directly observable and improvements in the management of high-risk cases can be attributed to the performance of the program. The program collected data on the prevalence (prev) and CFR for the two most common causes of maternal death at RRH, OH and HDoP, which accounted for 59% of the maternal deaths during the intervention. The hypothesis is that the QI intervention led to improvements in these two causes of mortality in particular through improved preparation, communication, and protocol adherence. Maternal deaths caused by other etiologies were considered stable in this analysis. Applying the steady-state assumption for the CFR, we compared observed outcomes to predicted fatalities based on CFRs for these two common conditions in 2007, which can be considered the reference year.

$${CFR}_{condition}^{Year}=\frac{Number\;of\;deaths\;linked\;to\;condition\;in\;a\;year}{Number\;of\;patients\;presenting\;with\;condition\;in\;that\;year}$$

$$Deaths\;Averted=\sum_{2011}^{2008}{Prev}_{HDoP}^{n}\times ({CFR}_{HDoP}^{2007})-{Deaths}_{HDoP}^{n}+{Prev}_{OH}^{N}\times \;({CFR}_{OH}^{2007})-{Deaths}_{OH}^{n}-{Deaths\;}_{Other}^{n}$$

Following calculation of the deaths avoided, the DALYs avoided were calculated based on the same set of assumptions described above, for determining the DALY burden of maternal deaths and intrapartum stillbirth death.

Finally, we are unable to calculate a CFR for stillbirths, because we do not have data on the prevalence of fetal distress or birth asphyxia. Therefore, for stillbirths, we first assumed that the counterfactual stillbirth rate (SBR) would remain steady at 9/1,000 life births as seen in 2007. The avoided stillbirths were then obtained by subtracting the number of intrapartum stillbirths observed from this counterfactual number.

### Assessing the cost-effectiveness of treatment

The relative cost effectiveness of the QI program was determined using the ICER, which shows the program cost effectiveness as measured in estimated attributable DALYs averted due to the program.

$$ICER=\frac{{Cost}_{QI}-{Cost}_{Null}}{{DALY}_{QI}-{DALY}_{Null}}$$

The WHO-CHOICE project used the work of the Commission of Macroeconomics and Health[47, 48] to argue that interventions having an ICER less than the country-specific GDP per capita can be considered highly cost effective. If the ICER is less than three times the GDP per capita, the intervention is deemed cost effective. These thresholds, while somewhat arbitrary and atheoretic, have been used by other studies in maternal health, and more generally in a range of assessments of the cost effectiveness of health interventions.[17,40, 49] The GDP per capita in Ghana averaged over 2007–2011 was $1268; this represents the benchmark for a highly-cost effective intervention in this study. [50]

### Sensitivity analysis

Assumptions made in the analysis were subjected to sensitivity analysis using Monte Carlo simulations run in Crystal Ball (Oracle, Redwood Shores, CA), which is an add-in program to Microsoft Excel (Microsoft, Redmond, WA). The assumptions made for calculating DALYs using discounting and standard formulas were varied as uniform distributions around high and low estimates as shown in Table 2. The performance of the program and changes in the acuity of disease were varied around a normal distribution for each parameter. Using 10,000 trial simulations, 95% confidence intervals for maternal and fetal deaths prevented, and cost effectiveness outcomes, were obtained.

## Results

### Estimating DALYs averted through the Kybele-GHS partnership

**Fig 2:**
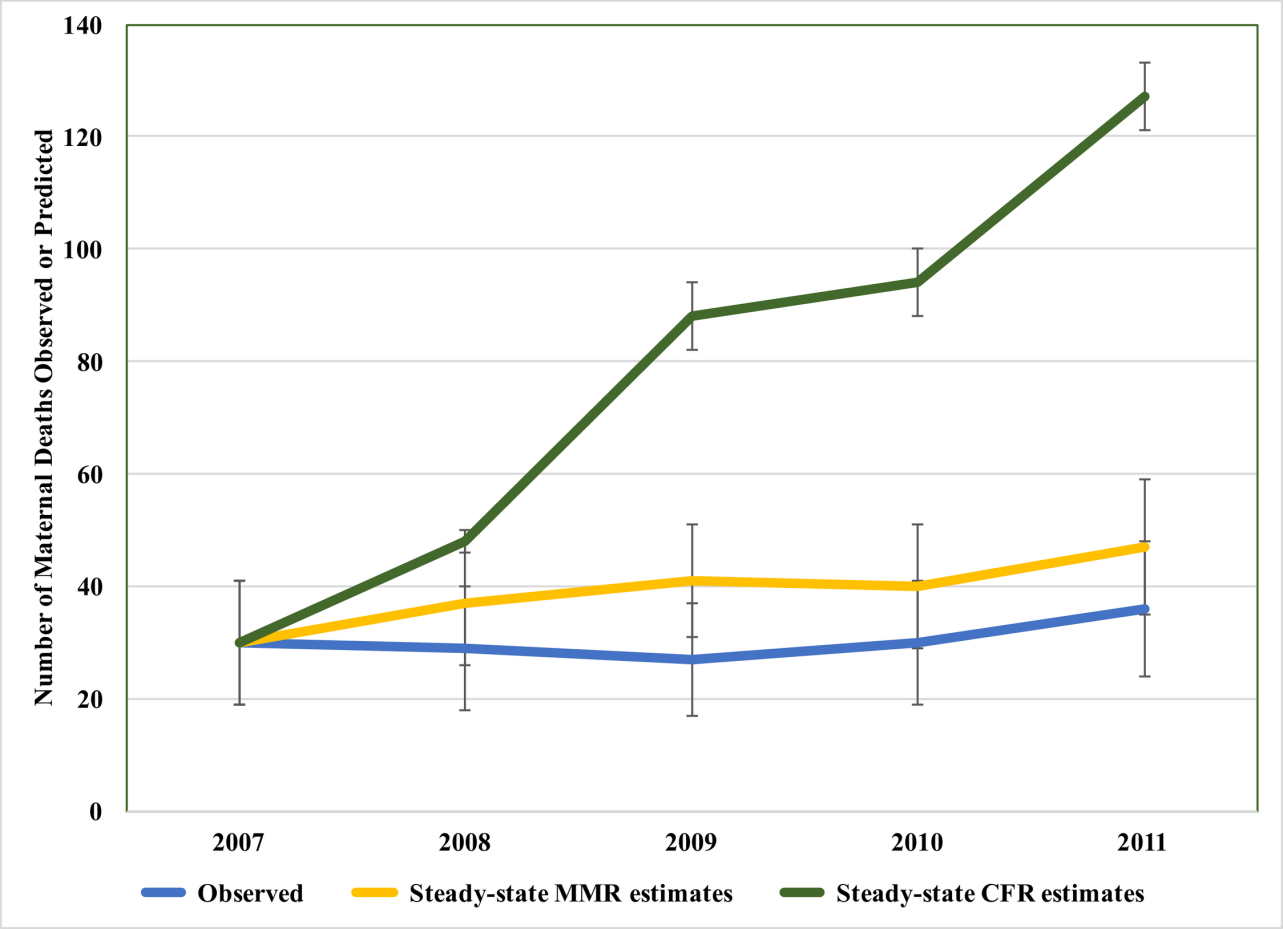
Maternal deaths predicted to have occurred by assumption. MMR, maternal mortality ratio (maternal deaths per 100,000 live births); CFR, case-fatality rate.

**Fig 3:**
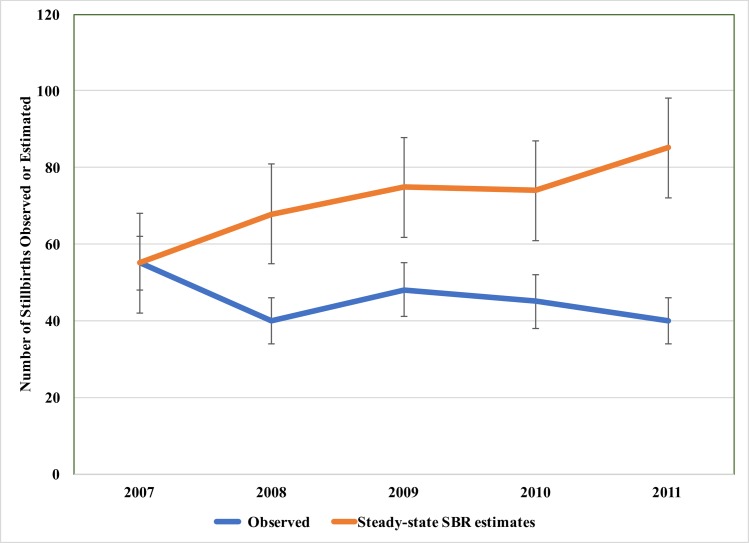
Stillbirths predicted to have occurred by assumption. SBR, stillbirth rate (intrapartum stillbirth per 1000 live births).

Baseline delivery and outcome data are presented in Table 3. During the intervention period, MMR decreased by 22% from 496 to 385 maternal deaths per 100,000 live births. Based on a steady-state assumption for the MMR, 43 maternal deaths were averted with an observed annual rate of mortality reduction ranging from -6 to -24% over five years. Because significant changes in volume, acuity, and CFR occurred over the course of the partnership, however, the estimated number of deaths prevented using a steady-state MMR may be underestimated (Fig 2). Applying a steady-state CFR instead, we predict that 245 maternal deaths were averted.[15] Ghana as a country experienced an annualized rate of change (ARC) of -3.6% over the course of the intervention.[51] Accounting for this trend in improvements occurring in the country would thus explain only 9 of these maternal deaths averted. The intrapartum stillbirth rate decreased 52%, and an estimated 129 stillbirths were prevented (Fig 3).

**Table 3:** Baseline data for the Kybele-GHS partnership.

Parameter	2007	2008	2009	2010	2011
**Total Deliveries**	6049	7465	8230	8133	9357
**Observed MMR (per 100,000 live births)**	496	388	328	369	385
**Number of Obstetric Hemorrhage cases (Prevalence, %)**	54 (0.9)	97 (1.3)	321 (3.9)	342 (4.2)	487 (5.2)
**Number of Hypertensive disorders of pregnancy cases (Prevalence, %)**	321 (5.3)	582 (7.8)	996 (12.1)	1033 (12.7)	1357 (14.5)
**OH CFR (%)**	14.8	5.1	1.9	2.0	1.6
**HDoP CFR (%)**	3.1	1.3	1.1	1.1	1.1
**Observed number of maternal deaths**	30	29	27	30	36
**Due to OH**	8	5	6	7	8
**Due to HDoP**	10	8	11	11	15
**Due to Other causes**	12	16	10	12	13
**Estimated number of maternal deaths based on steady-state MMR assumptions**	30	37	41	40	46
**Maternal deaths prevented due to steady-state MMR assumptions**	0	8	14	10	10
**Estimated number of maternal deaths based on steady-state CFR assumptions**	30	47	93	97	130
**Maternal deaths prevented due to steady-state CFR assumptions**	0	18	66	67	94
**Observed number of intrapartum stillbirths**	55	40	48	45	40
**Observed SBR (per 1,000 live births)**	9.0	5.4	5.8	5.5	4.3
**Estimated number of stillbirths based on steady-state SBR assumptions**	55	68	75	74	85
**Stillbirths prevented based on steady-state SBR assumptions**	0	28	27	39	45

Years of life lost (YLL) per maternal death were calculated as described above, with and without discounting and age-weighting. The age-specific life expectancy for a woman dying in pregnancy ranged from 54 to 29 depending on her age when she died. Using the proportions of deaths occurring in each age group, the total YLLs for an average maternal death were found to be 28.6 (43.4 undiscounted). Estimating the years of life spent disabled requires estimation of an average disability weight due to disability, as well as the duration of that disability. Given these assumptions, we obtained an additional 17.18 YLD (26.0 undiscounted) averted for every maternal death prevented. For stillbirths, the calculated YLL was 31.4 (61.2 undiscounted) years.

In total, therefore, the DALY per maternal death was 45.8 (69.4 undiscounted). Base calculations show that 236 maternal deaths were averted by the program leading to 10,838 DALYs avoided. If the 129 prevented stillbirths are also included, an additional 4,038 DALYs were averted.

### Cost-effectiveness analysis

The cost effectiveness of the Kybele-GHS partnership can be compared to the GDP thresholds for cost-effectiveness. Including all 14,876 DALYs averted, the ICER was found to be $158 ($129-$195). This is 8 times lower than the 2007–2011 Ghanaian GDP per capita, and indicates that the intervention was highly cost-effective. Without taking stillbirth into account, the program was still very cost effective with an ICER of $218 ($179–282) for 10,838 maternal DALYs averted. With the more conservative steady-state MMR assumption, and not considering stillbirths, yields an ICER of $1212, which remains below the cost-effectiveness threshold.

### Sensitivity analysis: Considering the changing environment

**Fig 4:**
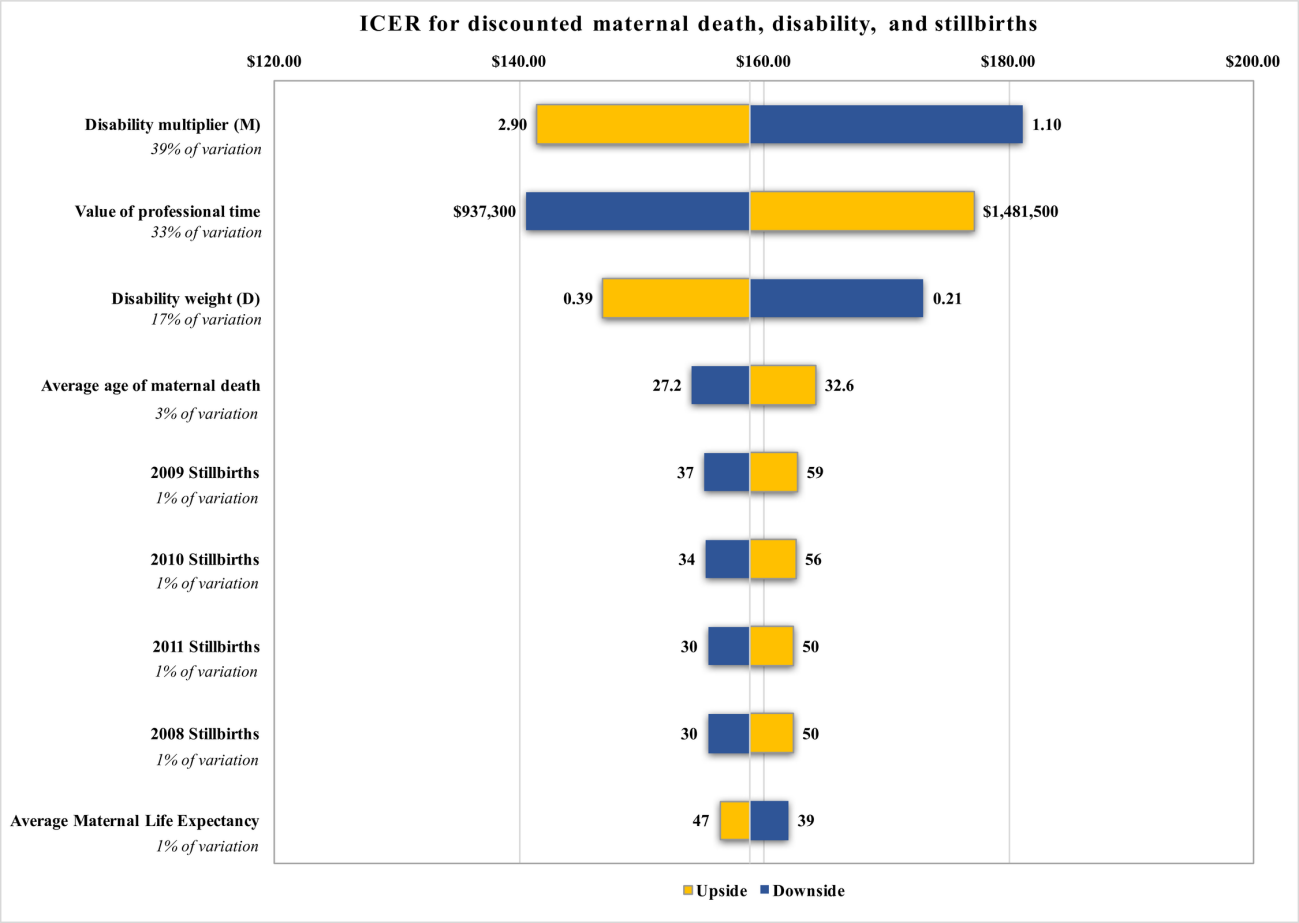
Tornado Chart of ICER for discounted calculations of maternal deaths, disability, and stillbirths showing all variables contributing ≥ 1% of the variation in the estimates. ICER, incremental cost-effectiveness ratio; Upside, this assumption makes the program more cost effective; downside, this assumption makes the program less cost effective.

This study is unique in that it reports on the results from a completed intervention, but several assumptions needed to be made, notably regarding the unobserved counterfactual. Table 2 describes the model parameters and describes how uncertainty was handled for each of these. We varied performance as a normal distribution centered about an observed mean to account for uncertainty. We used Monte Carlo simulations to take random draws from the specified parameter distributions in order to derive the credibility estimates presented in Table 4. The tornado chart in Fig 4 further displays the 5^th^ and 95^th^ percentile estimates for each assumption and how that variation individually affects the estimate of the ICER. The most significant assumption is the disability multiplier, i.e. the assumption that for every maternal death prevented, 1 to 3 women would also not suffer disabilities. This assumption accounts for 39% of the variation in the ICER estimate. The next most significant assumption was the value of professional time that was estimated as part of the budget. Centered around a base estimate of $1.21 million, changes by ± $0.27 million alter the ICER by $18/DALY avoided, which corresponds to 22% of the variation observed. The disability weight assumption ranging from 0.2–0.4 contributes an additional 12% of the variation, but is based on the values used in the Global Burden of Disease[43]. Finally, variability in the average age of maternal death contributes 3% of the variation.

**Table 4:** Sensitivity analysis*.

Parameter	DALYs	95% CI	CER	95% CI
**Discounted maternal deaths and disability**	10,838	8,679–13,561	$218	$170-$282
**Discounted maternal death, disability and stillbirths**	14,876	12,588–17,637	$158	$129-$195
**Undiscounted calculations maternal deaths and disability**	16,422	12,910–20,776	$144	$111-$189
**Undiscounted maternal deaths, disability and stillbirths**	24,300	20,477–28,844	$97	$79-$120

*Based on model estimates of 236 (±5) maternal deaths and 129 (±13) stillbirths prevented

## Discussion

### Main findings

We present the cost-effectiveness of a systems-based QI intervention using prospectively collected financial records and outcome data. This study serves to provide accountability to Kybele for their actions, and to direct inform the planning of other similar interventions. Our analysis shows that with $2.4 million invested, including $1.2 million worth of dontated professional time, the Kybele-GHS partnership was able to prevent between 43 and 236 maternal deaths and 129 intrapartum stillbirths. This amounts to 14,876 discounted DALYs (24,300 undiscounted DALYs), and leads us to conclude that the intervention was highly cost-effective, with an ICER of $158 ($129-$195) 2015 USD. Sensitivity analysis strengthens this conclusion; variation in key assumptions resulted in ICERs ranging from $79 to $282.

It is not typically feasible to systematically control interventions such as this, which makes attribution of causality difficult. It is likely that the “no intervention” counterfactual would have involved increasing mortality rates as a consequence of the increasing prevalance in OH and HDoP, and that the increase would have been on the order of 236 maternal deaths. Such a counterfactual would have led to an MMR similar to those observed in other referral hospitals in Ghana, which ranged from 913–1004 maternal deaths per 100,000 live births.[52, 53] Our study shows that RRH was able to achieve significantly lower CFRs than these other hospitals over the intervention period.

The GHS and RRH continued to provide care through the national health insurance scheme and patient fees. It is likely that these charges would have been present without the partnership as the caseload at the hospital increased and in accordance with the treatments administered. The QI intervention was intended to promote higher quality and more efficient care, which could translate into lower costs. For example, the provision of more rapid treatment for conditions such as OH or HDoP, may help mitigate expensive treatments such as blood transfusions and intensive care admissions. We cannot, however, assess how the costs of clinical care changed at RRH during the course of the intervention.

Cost-effectiveness is not routinely reported by organizations working to improve MFH, so our analysis addresses an important knowledge gap. To the extent possible in this retrospective analysis, the CHEERS guidelines[54] for reporting cost-effectiveness were followed. This is a single study-based estimate that therefore serves as a valuable source for understanding the potential cost of long-term coaching, leadership development, and quality improvement measures that others might attempt in similar facilities. Nonetheless, the incremental effectiveness of such interventions may vary considerably as a function of the heterogeneity of health systems and demand-side influences, and should be the subject of additional future studies.

One important issue that this analysis ignores is that Ridge Hospital serves as a referral hospital, and since the Kybele–GHS partnership was initiated, outreach to referral centers, educational modules and strengthening of protocols has also occurred. The benefits of this outreach, which may be significant, were not included here. Further analysis at the regional levels is therefore urgently needed to deepen our understanding of the value of those aspects of the program.

### Strengths and limitations

This study has several advantages. It retrospectively analyses a real-world intervention rather than relying on hypothetical constructs developed prospectively. This project was established as a shared initiative between Kybele and the GHS. By achieving local buy-in from the GHS, Kybele was able to create a sense of partnership that allowed prioritization and access to decision makers. The project provided the development of leadership and QI skills to sustain healthcare improvements at Ridge and at other sites within the GHS. Cost-sharing by the government indicated commitment to the program, and increases the likelihood of its longevity.

There are also weaknesses present that are important to acknowledge. The project was conducted using volunteer time donated by foreign experts. This makes the pecuniary cost of the program modest, and required us to estimate the value of the donated professional time. Relatedly, most hospitals in low-income settings will not have ready access to skilled personnel able to support similar programs. The QI intervention was extremely time intensive and may not be reproducible in other settings due to constraints on manpower and funding. Third, the study was implemented in a single hospital. The estimates of impact on maternal and fetal mortality are based on pre- and post-intervention comparisons; as such, the precise number of deaths averted cannot be known, especially in light of the significant changes in the number of births and disease acuity observed at the hospital over time. These changes were not anticipated at the project outset and may have resulted from changes in referral patterns in Accra, insurance incentives promoting institutional delivery, and community recognition of improving care at RRH. Considering the facilities around the world in which women deliver, RRH is a moderately well-staffed and equipped hospital. This limits external validity as the cost effectiveness of this type of intervention would vary across hospital and clinic settings. Finally, it is difficult to estimate the effectiveness of interventions addressing maternal health without also considering neonatal health as well, which were not carefully followed and documented.

Assessing the long-term effectiveness of a program also requires careful consideration of whether the changes induced can be sustained over time. It is our hope that the coaching and leadership development model helped to change the culture at RRH in such a way that improvements can now be led by local providers with gradually decreasing intervention from Kybele. During the intervention, leaders and staff who displayed a high level of ability and interest in the QI process were identified as “clinical champions.” Since 2011, much of the work at RRH has focused on enabling these champions to lead and sustain improvement. Frequently, they have been asked by the GHS to provide trainings at other facilities. Assessing the longer-term effects of the intervention will be the subject of future research.

### Interpretation

As governments continue to emphasize and incentivize institutional delivery as part of their MFH improvement packages, the need for high-quality CEmOC will continue to rise. We report that it may be highly cost-effective to support CEmOC with QI methodologies led by visiting consultants. Over time, the goal is to develop enough internal capacity to lead these efforts in LMICs. We believe that NGOs can play an important role in building that capacity by partnering with ministries of health.

## Conclusion

The Kybele-Ghana Health Service Partnership was able to reduce maternal and fetal mortality over the course of five years using quality improvement methodologies. Applying an approach that predicted the impact of this intervention based on an assumption of steady-state CFRs, it was estimated that the program prevented 236 (±5) maternal deaths and 129 (±13) stillbirths, which amounted to 14,876 DALYs averted. The best estimate of the ICER was therefore found to be $158, a value well below the very cost effective thresholds based on per capita GDP in Ghana ($1,268 USD). This study supports the hypothesis that collaborative partnerships using quality improvement methodologies can produce cost-effective outcomes. Additional similar experiences need to be studied in other settings, but it seems likely that quality improvement interventions that address systems issues in other resource-constrained settings and at larger scale will often be similarly cost-effective.
